# Exposed nucleoprotein inside rabies virus particle as an ideal target for real-time quantitative evaluation of rabies virus particle integrity in vaccine quality control

**DOI:** 10.1371/journal.pntd.0013077

**Published:** 2025-05-30

**Authors:** Jia Li, Yuhang Yang, Kuangrou Jia, Zhigao Zhang, Xiangmin Zhai, Yue Cao, Xinbiao Huang, Shouchun Cao, Yingsong Wu, Guanfeng Lin

**Affiliations:** 1 National Institutes for Food and Drug Control, Beijing, China; 2 State Key Laboratory of Drug Regulatory Science, Beijing, China; 3 Institute of Antibody Engineering, School of Laboratory Medicine and Biotechnology, Southern Medical University, Guangzhou, China; 4 Medical Big Data Center, Guangdong Provincial People’s Hospital (Guangdong Academy of Medical Sciences), Southern Medical University, Guangzhou, Guangdong Province, China; The University of Kansas, UNITED STATES OF AMERICA

## Abstract

Currently, the integrity of rabies virus particle in quality control can only be assessed through electron microscopy. However, its time-consuming nature, operational complexity, limited accuracy and lack of quantification capability no longer meet the needs of modern vaccine production. Based on this, we developed a time-resolved fluoroimmunoassay (TRFIA) to enable real-time quantitative analysis of rabies virus particle integrity in human rabies vaccine, by detecting exposed nucleoprotein. Monoclonal antibodies against rabies glycoprotein and nucleoprotein were prepared using the classical hybridoma technology with the aim of constructing a novel detection approach for assessing particle integrity. A monoclonal antibody against rabies glycoprotein was immobilized on microplate wells to capture rabies virus particles, while a labeled antibody against the nucleoprotein served as the signal tracer. Multiple types of vaccine samples were analyzed to evaluate the effectiveness and accuracy of the developed TRFIA, combined with various virus particle destruction methods to validate its capability to assess particle integrity. The validation results were consistent with those obtained from electron microscopy. Therefore, this novel TRFIA offers a promising solution to address existing gaps in current analytical methods, enabling straightforward real-time, and quantitative evaluation of rabies virus particle integrity in the laboratory.

## Author summary

Vaccination is widely recognized as the most effective and safest strategy for rabies control, serving as a cornerstone of prevention efforts. Since all current commercialized rabies vaccines are inactivated virus formulations, preserving the structural integrity of viral particles in the final product is a key indicator of production quality; thus, particle integrity analysis constitutes a critical component of vaccine quality control. Traditional electron microscopy-based methods used for assessing particle integrity have notable limitations and fail to meet the demands of contemporary vaccine production quality standards. To address this, we developed a time-resolved fluoroimmunoassay (TRFIA) that enables real-time, quantitative analysis of rabies virus particle integrity in human vaccines, through detection of exposed nucleoprotein within disrupted particles. This method may help bridge the current gap in quality control by enabling simple and reliable real-time quantitative evaluation of rabies virus particle integrity.

## 1. Introduction

Rabies is a severe viral zoonosis caused by the rabies virus (RV), primarily transmitted via the saliva of infected animals through bites or scratches[1]. RV infects the central nervous system and causes acute encephalitis, with an almost 100% mortality rate once clinical symptoms emerge[2]. Historically, rabies has been a significant cause of mortality in many parts of the world. According to the World Health Organization (WHO), more than 59,000 people die from rabies annually, and approximately 95% of these fatalities occur in Africa and Asia[3]. Most victims are children under the age of 15 who get infected typically through dog bites[4]. Hence, rabies remains a major public health issue that requires sustained global attention and coordinated control efforts[5].

Vaccination is widely acknowledged as the most effective and safest strategy for rabies control, forming the cornerstone of prevention programs[6,7]. Given the critical role of rabies vaccines in disease prevention, it is essential to upgrade key quality control techniques to ensure the consistent production of high quality vaccines[8]. Since all current commercialized rabies vaccines are inactivated rabies virus formulations, particle integrity serves as a key indicator of production quality[9,10]. Thus, evaluating the structural integrity of rabies virus particles is a fundamental component of vaccine quality control[9].

Currently, assessment of rabies virus particle integrity relies exclusively on electron microscopy[11]. However, this technique has several limitations, such as inability for real-time detection, high costs, technical complexity, low accuracy, and limited quantifiability[12],. The quality control of rabies vaccines involves a number of immunological tests, such as assessment of glycoprotein content, bovine serum albumin residues, and host cell protein residues[13–15]. The development of innovative immunoassay-based technologies for particle integrity assessment would significantly improve the efficiency and robustness of the quality control system.

Time-resolved fluoroimmunoassay (TRFIA), since its initial application in rabies vaccine quality control, has proven to be an excellent analytical platform due to its outstanding specificity, high accuracy, wide linear range and operational simplicity[13,16]. Compared with the enzyme-linked immunosorbent assay (ELISA), which is commonly used for vaccine quality control, TRFIA exhibits markedly enhanced sensitivity, accuracy, and detection range[13,17,18].

In this study we developed a novel TRFIA for real-time quantitative assessment of rabies virus particle integrity based on the detection of exposed nucleoprotein. A monoclonal antibody against rabies virus glycoprotein was immobilized on microplate wells to capture rabies virus particle, while a labeled anti-nucleoprotein antibody served as a signal tracer. Multiple vaccine samples were used to evaluate the effectiveness and accuracy of the present TRFIA, and validation results aligned well with those from electron microscopy. These findings confirmed that exposed nucleoprotein in rabies virus particle, as an ideal target, can be used for real-time quantification of rabies virus particle integrity in quality control. In addition, the proposed TRFIA may address current gaps in quality control analytical methods by enabling straightforward, real-time, and quantitative evaluation of viral particle integrity in laboratory settings.

## 2. Materials and methods

### 2.1 Ethics statement

This study had been approved by the Laboratory Animal Ethics Committee of Southern Medical University. The care and use of the animals conform to the Institutional Animal Ethics Committee guidelines.

### 2.2 Virus, cell, animal and samples

The inactivated rabies virus, collaborative calibration of the 9th national standard for human rabies vaccine (CTN strain, batch number: 250009–201909, potency: 11.4 IU/mL), vero cell protein standard (batch number: 250022–201901, concentration: 2.6 μg/mL), primary hamster kidney cell protein standard (batch number: 250024–201901, concentration: 4 μg/mL) and human rabies vaccine samples were obtained from National Institutes for Food and Drug Control (Beijing, China). Purified recombinant glycoprotein (G), purified recombinant nucleoprotein (N), Sp2/0 cell were stored at the Institute of Antibody Engineering, School of Laboratory Medicine and Biotechnology, Southern Medical University (Guangzhou, China). Female BALB/c mice (6–8 weeks old) used in this study were purchased from Experimental Animal Center of Southern Medical University (Guangzhou, China).

### 2.3 Reagents and instrumentation

Bovine serum albumin (BSA), human serum albumin (HSA), trehalose, gelatin, dextran-40, Freund’s complete adjuvant, Freund’s incomplete adjuvant, TMEMD, Tween-20, and glutaraldehyde were purchased from Sigma-Aldrich (St. Louis, MO, USA); Europium (Eu) labeling kit (YB-EU-1), rabies virus nucleoprotein analysis kit (YB-RVN-1), rabies virus glycoprotein analysis kit (YB-RVG-1), virus lysis buffer, and enhancement solution (YB-TRF-1) were sourced from Guangzhou You Bo Biotechnology Co., Ltd. (Guangzhou, China); Ultrafiltration centrifuge tubes were purchased from Millipore Corp. (Billerica, MA, USA); Protein purification column filler Sephadex G-50 was purchased from Amersham Pharmacia Biotech (Piscataway, NJ, USA). Ultra-pure water preparation system and ultramontane electronic balance were obtained from Sartoruis (Gottingen, Germany); Microplate thermostatic oscillator was purchased from Thermo Fisher Scientific (Waltham, MA, USA); DR6660 was purchased from Guangzhou DARUI Biotechnology Co., Ltd. (Guangzhou, China). All other chemicals and reagents used were of analytical grade and used without further purification.

### 2.4 Solutions

Coating buffer was 50 mM Na_2_CO_3_-NaHCO_3_ buffer (pH 9.6). Blocking solution was 50 mM Na_2_CO_3_-NaHCO_3_ buffer (pH 9.6) containing 1% BSA. Labeling buffer was 50 mM Na_2_CO_3_-NaHCO_3_ (pH 8.5) with 155 mM NaCl. Elution buffer was 50 mM Tris-HCl (pH 7.4) with 0.2% BSA and 0.9% NaCl. Standard buffer was 50 mM Tris-HCl (pH 7.8) containing 0.1% NaN_3_ and 0.2% BSA. Assay buffer was 50 mM Tris-HCl (pH 7.8) with 0.02% BSA, 0.05% Tween-20 and 0.05% NaN_3_. Washing buffer was 25 mM Tris-HCl (pH 7.8) with 0.9% NaCl and 0.06% Tween-20.

### 2.5 Preparation of monoclonal antibody (Mab)

Monoclonal antibodies were prepared following standard protocols by immunizing 6–8 weeks old female BALB/c mice with inactivated rabies virus (PM strain)[19]. Inactivated rabies virus samples were emulsified with Freund’s adjuvant and administered to mice via subcutaneous multipoint injection. Three weeks later, a booster injection was performed using the same method, resulting in a total of two immunizations for each mouse. One week after the booster, blood was collected from the tail vein to evaluate antibody titer. Splenocytes isolated from those BALB/c mice were fused with Sp2/0 cells to prepare the hybridoma. The hybridoma cells secreting monoclonal antibodies against G and N of rabies virus were screened via indirect TRFIA assay with purified recombinant G and N, and the monoclonal hybrids were selected after two rounds of single cell cloning. Each positive hybridoma cell line was intraperitoneally injected into mice to produce ascites. To obtain monoclonal antibodies, the recovered ascites was purified using proteinG affinity chromatography.

### 2.6 Immobilization of capture MAb

Each well of the standard, bare 96-well plates was coated with anti-G MAb diluted in 100 μL coating buffer and incubated at 4 °C for 12 h. After three washes, blocking solution (250 μL) was added to the well and incubated at 37 °C for 1 h to block the coated surface. Finally, the 96-well plates were dried under high vacuum, and stored at -20 °C in a sealed plastic bag containing desiccant.

### 2.7 Labeling of MAb

According to the protocol provided by the Eu^3+^-labeling kit, anti-N MAb was labeled with Eu^3+^-chelates. Briefly, 400 μg of Eu^3+^-chelates was gently mixed with 2 mg of anti-N MAb in 200 μL labeling buffer and incubated at room temperature for 18 h with continuous gentle shaking. Then the mixture was separated by a Sephadex G-50 column. Purified Eu^3+^-MAb conjugates were eluted with descending elution buffer, and collected (1.0 mL per fraction). The protein concentration of Eu^3+^-MAb conjugates in collected fraction was measured using the Pierce BCA Protein Assay Kit. The fluorescence of Eu^3+^-MAb conjugates diluted with enhancement solution (1:100) was detected in microtitration wells (200 μL per well). Fractions from the first peak with the highest Eu^3+^ count were pooled and further characterized. After dilution with elution buffer containing 0.2% BSA as a stabilizer, the purified conjugates were rapidly lyophilized under high vacuum and stored at -20 °C.

### 2.8 Preparation of standards

The collaborative calibration of the 9th national standard for human rabies vaccine (assigned a value of 1 EU/mL for exposed N protein per vial) was serially diluted with standard buffer to obtain calibration standards at the following concentrations: 0 mEU/mL, 2.5 mEU/mL, 12.5 mEU/mL, 25 mEU/mL, 50 mEU/mL and 125 mEU/mL, respectively. Then standards were divided into 1 mL/bottle, lyophilized and stored at 4 °C, and re-dissolved with 1 mL pure water prior to use.

### 2.9 Immunoassay design

**Fig 1:**
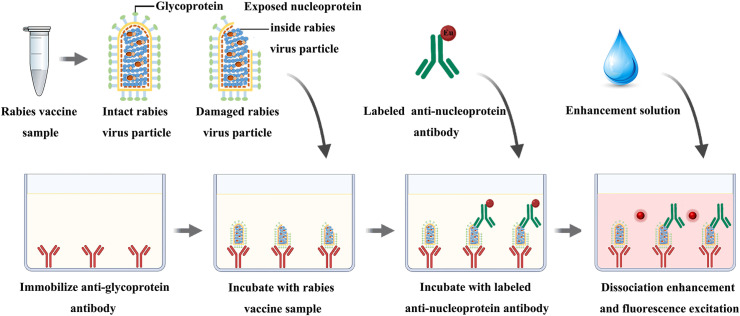
Schematic diagram of this present TRFIA for real-time quantitative evaluation of virus particle integrity.

As shown in Fig 1, the assay was performed using the two-step procedure. Following the immobilization and antibody labeling protocols described above, 100 μL of standards or samples was added into each well and incubated at room temperature for 1 h with gentle shaking on an oscillator. Then, 100 μL of assay buffer containing Eu^3+^-labeled anti-N MAb was added into each well after washing. After one-hour incubation with continuous slow shaking, a sandwich complex was formed in each well by the immobilized MAb, samples or standards, and the Eu^3+^-labeled MAb. The wells were then washed for 6 times and filled with 100 μL enhancement solution. The plates were shaken for 5 min at room temperature and then the fluorescence intensity was measured using a DR6660 equipped with filters for Eu^3+^ (excitation wavelength, 340 nm; emission wavelength, 615 nm; delay time,0.40 ms; window time, 0.40 ms; cycling time, 1.0 ms). Samples exceeding the current TRFIA detection limit were diluted using assay buffer to ensure measurements within the linear range.

### 2.10 Optimization and validation

#### 2.10.1 *Optimization of antibody combination.*

The optimal antibody pair was identified by using a square matrix method. Rabies vaccine (100 μL/well) was added to the wells coated with different anti-G MAb and incubated for 1h at room temperature with shaking on an oscillator. After washing, each Eu^3+^-labelled anti-N MAb was diluted 1:500 with assay buffer, and 100 μL was added to the corresponding wells. Finally, the fluorescence value was measured to determine the optimal antibody combination.

#### 2.10.2 *Optimization of capture antibody concentration.*

The anti-G MAb was diluted to six different concentrations (0.5, 1, 3, 5 and 8 μg/mL) using coating buffer to determine the optimal concentration for capture antibody. Other parameters were held constant (detection antibody 1:400 dilution, 1-hour incubation).

#### 2.10.3 *Optimization of labeled antibody dilution.*

The Eu^3+^-labelled anti-N MAb was diluted to six different dilutions (1:50, 1:100, 1:200, 1:400, 1:800 and 1:1600) to determine the optimal dilution factor. Other parameters were held constant (3 μg/mL of capture antibody and 1 hour incubation).

#### 2.10.4 *Optimization of reaction Time.*

Incubation durations (20, 40, 60, 80 minutes) were evaluated to optimize antigen-antibody binding kinetics using 3 μg/mL of capture antibody and 1:200 of the labeled antibody.

#### 2.10.5 *Verification of design concept.*

The conceptual basis of the current TRFIA detection mode is that when the rabies virus particles are damaged by external factors and the outer membrane is disruted, the N inside the particles will be exposed. Within a certain range, the amount of exposed N in rabies virus particles is inversely correlated with the integrity of rabies virus particles in vaccine samples containing predominantly intact virions. When the extent of viral particle fragmentation is excessive, the exposed N in rabies virus particles will become unbound in solution and can no longer be captured and detected by this present TRFIA. Finished vaccine samples (PM strain) were treated with different concentration of virus lysis buffer to verify this design concept by using this present TRFIA (coated G-Mab & labled N-MAb), a commercial glycoprotein analysis kit (coated G-Mab &labled G-MAb) and a commercial nucleoprotein analysis kit (coated N-Mab & labled N-MAb).

#### 2.10.6 *Standard curve.*

Ten replicate wells were set up for each concentrations of the standard and the corresponding fluorescence values were measured using the present TRFIA method. Logarithmically transformation was applied to the data for curve fitting. Standard curves were obtained by linear regression of the logarithm of the fluorescence intensity value (Y) against the logarithm of the sample concentration (X): LogY = A × LogX + B.

#### 2.10.7 *Assay sensitivity.*

The blank standard (0 mEU/mL) was repeatedly detected 20 times in accordance with the TRFIA protocol, and the mean and standard deviation (SD) of the fluorescence signal values were calculated. Then the fluorescence value generated by mean+2 × SD was substituted into the standard curve equation, and the calculated concentration was defined as the detection limit of the present TRFIA.

#### 2.10.8 *Assay precision.*

The precision of this assay was evaluated based on intra-assay and inter-assay. Three quality control samples (5, 35, 75 mEU/mL) were measured 10 times per day for 3 consecutive days. The fluorescence signal values were recorded and analyzed. The inter-assay and intra-assay coefficient of variation (CV) were calculated as the standard deviation divided by mean.

#### 2.10.9 *Specificity assay.*

Sucrose, maltose, trehalose, gelatin, dextran-40, Vero cell protein, primary hamster kidney cell protein, BSA and HSA, as conventional components of rabies vaccine, were used to assess the specificity of present TRFIA method. Rabies vaccine samples (PM strain) were spiked with 2000 ng/mL of each of the above components and tested according to the TRFIA procedure. The measured concentrations were compared with the theoretical values to assess specificity (recovery rate = detection value/theoretical value×100%).

#### 2.10.10 *Assay* accuracy.

Multiple vaccine samples (PM strain) with varying degrees of particle integrity following the same manufacture process were analyzed using a commercial rabies virus glycoprotein detection kit. The glycoprotein values of each sample were adjusted to similar levels, and those samples were then used to evaluate the effectiveness and accuracy of the present TRFIA. Also, finished vaccine sample (PM strain) were treated with different time of ultrasonic disruption, different numbers of repeated freeze-thaw treatments (frozen at -80 °C, dissolved in 37 °C water), various durations of heat treatment at 50 °C and different concentrations of virus lysis buffer to artificially compromise the integrity of the rabies virus particle for accuracy analysis of the present TRFIA. All analyses in this step were performed in duplicate to ensure the credibility of the results.

#### 2.10.11 *Comparison with electron microscope observation.*

Fresh purified inactivated virus particle samples were analyzed using both electron microscopy and the present TRFIA, comparing a group subjected to 10 repeated freeze-thaw treatment and an untreated control group. The results from both analyses were compared to assess the feasibility and accuracy of the present TRFIA. Electron microscope was conducted as follows: the rabies virus particle sample was pipetted onto a copper screen and the excess liquid was aspirated from the edge of the screen with a filter paper after 2 min. A few drops of 3% phosphotungstic acid (pH 7.0) were applied to the copper grid, incubated for 2 min, and the excess stain was removed from the edge of the grid with a filter paper. Drops of pure water were added to the copper grid, and the excess was removed after 1 min, and the copper grid was allowed to air dry for electron microscopic observation.

### 2.11 Statistical analyses

Statistical analyses were performed using Statistical Product and Service Solutions (SPSS) software (version 20.0, SPSS Inc., Chicago, IL). A two-tailed test was applied for statistical analysis in all tests with alpha level set at *α* = 0.05. A *P* value of less than 0.05 was considered statistically significant.

## 3. Result

### 3.1 Antibody screening and identification

The serum potencies of mouse immunized with purified inactivated PM strain rabies virus are shown in S1 Table. The results of indirect TRFIA identification of anti-G or N positive hybridoma clones are presented in Table 1. Finally, three anti-G MAbs (RG10, RG18 and RG56) and four anti-N MAbs (RN03, RN19, RN42 and RN44) were obtained using hybridoma technique.

**Table 1:** Results of indirect TRFIA identification of anti-G or N positive hybridoma clones.

Antigen	ID of hybridoma clone	Fluorescence intensity
Purified recombinant G	RG10	185452
	RG18	164421
	RG56	191421
	Blank control (RPMI 1640 Medium)	1245
Purified recombinant N	RN03	178441
	RN19	192145
	RN42	225411
	RN44	184541
	Blank control (RPMI 1640 Medium)	1098

### 3.2 Screening of antibody combination

**Fig 2:**
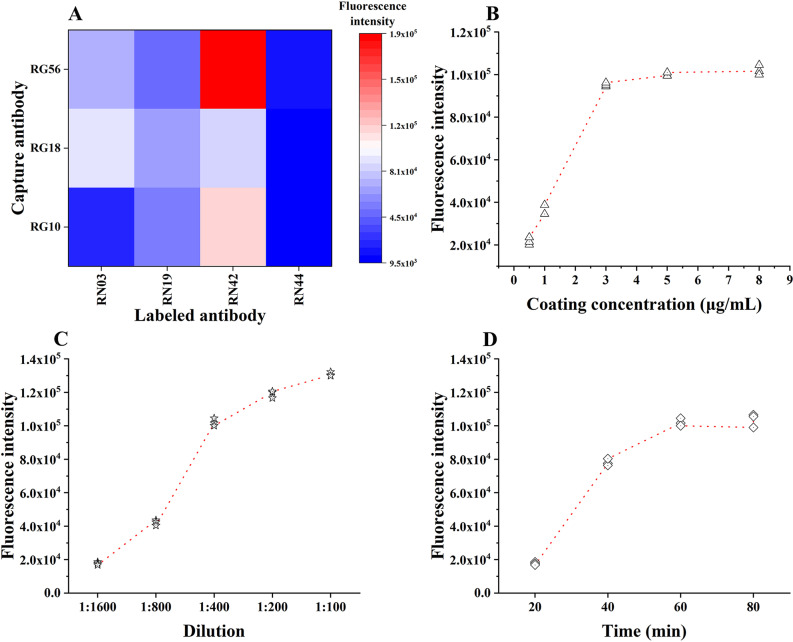
Optimization of the reaction system. A: Screening of antibody combination; B: Selection of the optimal capture antibody concentration; C: Selection of the optimal labeled antibody dilution. (D): Selection of the optimal reaction time.

Three anti-G MAbs (RG10, RG18 and RG56) and four anti-N MAbs (RN03, RN19, RN42 and RN44) identified in the previous step were used to screen for the optimal antibody combination using the square matrix method. As shown in Fig 2A, the fluorescence signal value was the highest when RG56 was used as the capture antibody and RN42 as the detection antibody. Therefore, this antibody pair was chosen for the following construction of this novel TRFIA. The corresponding detailed data are shown in S2 Table. In addition, specificities of RN42 and RG56 were identified by western-bolt using purified inactivated PM strain rabies virus (S1 Fig).

### 3.3 Optimization of the assay system

As depicted in Fig 2B, the fluorescence value did not significantly increase when the capture antibody concentration exceeded 3 μg/mL, indicating that the encapsulation concentration of the capture antibody was close to saturation. Therefore, 3 μg/mL was selected as the optimal capture antibody concentration. As shown in Fig 2C, the fluorescence value gradually increased as the dilution concentration of the detection antibody increased from 1:1600–1:100. When the dilution exceeded 1:400, the fluorescence increase became negligible. Considering cost efficiency, a dilution of 1:400 was selected as optimal for the labeled antibody. The fluorescence value significantly increased when the reaction time was extended from 10 min to 60 min in Fig 2D. However, no further increase in the fluorescence signal was observed beyond 60 min. Therefore, 60 min was selected as the optimal reaction time for subsequent experiments.

### 3.4 Verification of the design concept, standard curves, and limit of detection

**Fig 3:**
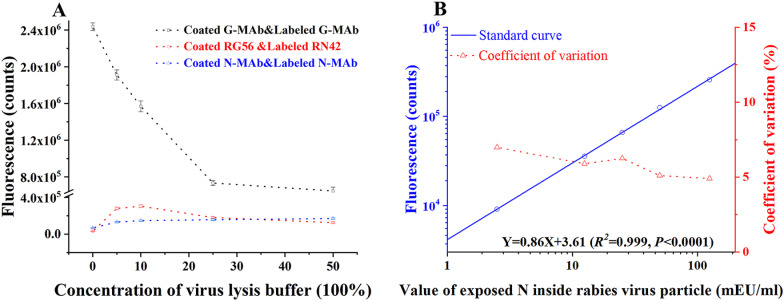
Verification of design concept and construction of standard curve. A: Analysis of signal trend change of three antibody combination modes for detecting vaccine product treated with virus lysis buffer of different concentration; B: Calibration curve and intra-assay precision profile of the novel TRFIA (each point was based on 10 replicates).

As revealed in Fig 3A, only the detected signal of this TRFIA (G-Mab & N-Mab) rose first and then fell. In contrast, the signal in the G-Mab & G-Mab mode continuously decreased, while that in the N-Mab & N-Mab mode kept rising. These results were in line with the expected effects of the cleavage treatment, and also preliminarily validated the design concept of this present TRFIA. Logarithmic and linear regression were used to determine the standard curve. As shown in Fig 3B, the following equation represents the best match calibration: LogY = 0.86 LogX + 3.61(*R*^*2*^ = 0.999), *P* < 0.001. As demonstrated in S8 Table, the detection limit was calculated by substituting the value of the background signal from the mean value plus 2 times the standard deviation into the standard curve equation, yielding a sensitivity of 0.08 mEU/mL. The linear range for the TRFIA technique was extended up to 125 mEU/mL.

### 3.5 Precision assay

The sample recovery rate was calculated by comparing the measured value of the sample with the theoretical value (recovery = measured value/theoretical value*100%). As shown in Table 2, all recovery rates ranged from 90% to 110%, indicating good accuracy. Both the inter-assay CVs and the intra-assay CVs were less than 10%, demonstrating good precision and meeting standard assay validation criteria.

**Table 2:** Precision assay of the present TRFIA.

	Sample	Theoretical value (mEU/mL)	Mean±SD (mEU/mL)	Recovery(%)	CV (%)
Intra-assay (n = 10)	Ⅰ	5	5.36 ± 0.33	107.2	6.15
	Ⅱ	35	36.62 ± 1.92	104.6	5.24
	Ⅲ	75	74.45 ± 3.87	99.2	5.20
Inter-assay (n = 30)	Ⅰ	5	4.84 ± 0.38	96.8	7.85
	Ⅱ	35	37.15 ± 2.59	106.1	6.97
	Ⅲ	75	72.35 ± 4.02	96.5	5.56

### 3.6 Specificity assay

As shown in Table 3, the recovery rates ranged from 90% to 110% in the presence of high concentration of sucrose, maltose, trehalose, gelatin, dextran-40, Vero cell protein, primary hamster kidney cell protein, BSA and HSA, demonstrating that the TRFIA exhibited good specificity.

**Table 3:** Specificity assay of the present TRFIA (n = 3).

Sample	Added concentration (ng/mL)	Theoretical value (mEU/mL)	Mean of measure value (mEU/mL)	Recovery (%)
Sucrose	2000	10	9.89	98.9
Maltose	2000	10	10.15	101.5
Trehalose	2000	10	10.25	102.5
Gelatin	2000	10	9.79	97.9
Dextran-40	200	10	9.97	99.7
Vero cell protein	2000	10	103.5	103.5
Primary hamster kidney cell protein	2000	10	9.86	98.6
BSA	2000	10	10.44	104.4
HSA	2000	10	10.38	103.8

### 3.7 Accuracy analysis

**Fig 4:**
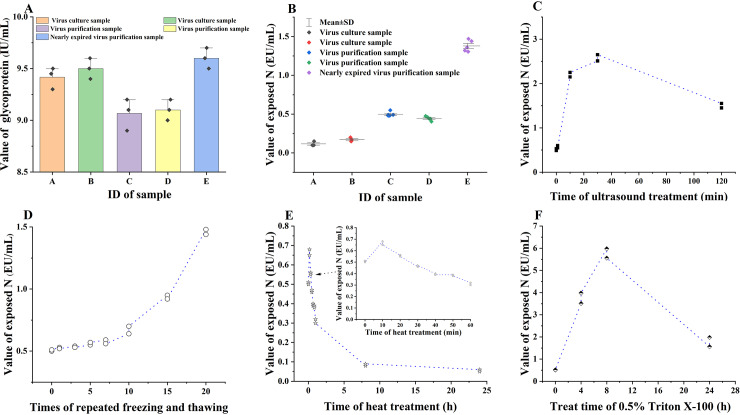
Accuracy analysis of the present TRFIA. A: Detection results of glycoprotein for multiple vaccine samples; B: Detection results of exposed nucleoprotein for multiple vaccine samples with different degrees of particle integrity; C: Detection of finished vaccine treated with different time of ultrasonic crushing; D: Detection of finished vaccine applying with different times of repeated freeze-thawing; E: Detection of finished vaccine applying with different time of heat treatment; F: Detection of finished vaccine applying with different concentration of virus lysis buffer.

As shown in Fig 4A and 4B, detection results of multiple vaccine samples with varying degrees of particle integrity but similar glycoprotein measurements were consistent with the expected integrity based on production stages. In the actual production process, the integrity of freshly harvested virus particle samples was the highest, followed by that of purified virus particle samples, and the integrity of nearly expired purified virus particle samples was the lowest. Our test results further validate this fact and attest to the accuracy of the present TRFIA. After subjecting the finished vaccine to ultrasonic crushing, repeated freeze-thawing, heat treatment and 0.5% Triton X 100, TRFIA accurately analyzed the changes of rabies virus particle induced by these treatments (Fig 4C, 4D, 4E and 4F).

### 3.8 Comparison with electron microscope observation

**Fig 5:**
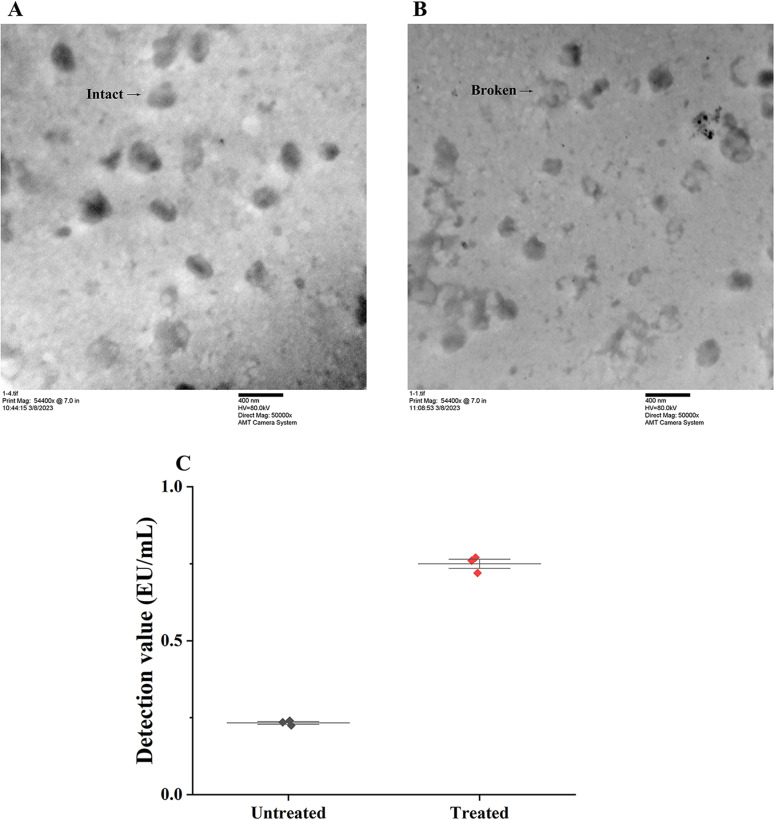
Comparison with electron microscope observation. A: Electron microscope observation result of purified inactivated virus particle sample; B: Electron microscope observation result of purified inactivated virus particle sample with 10 times repeated freeze-thaw treatment; C: Results of present TRFIA for assaying treated group and untreated control group of purified inactivated virus particle samples.

Fig 5A shows the state of the virus particles before repeated freeze-thaw treatment. It can be clearly seen that the virus particles after repeated freeze-thaw treatment particles exhibited more severe rupture, as electron microscopy images of the captured field of view revealed numerous virus particles fragments (Fig 5B). As shown in Fig 5C, the results of TRFIA were consistent with the electron microscopy observations, and the detection values of the treated group were significantly higher than those of the untreated group, indicating that the level of rabies virus particle integrity in the treated group was substantially lower than that in the untreated group. In addition, TRFIA provides stable and quantified data for the analysis of rabies virus particle integrity.

## Discussion

The primary advantage of using electron microscopy to evaluate the integrity of viral particles in rabies vaccine samples lies in its directness and visual accuracy [20]. However, the high procurement cost and complex operational training of electron microscopy necessitate that vaccine manufacturers send vaccine samples to specialized institutions for analysis. This introduces a risk of particle damage during transportation, as virus particles are inherently fragile. Moreover, sample fixation and negative staining required for electron microscope can also induce particle damage[20,21]. Therefore, it is difficult to distinguish whether the observed particle damage is inherent or introduced during transportation and sample preparation. In addition, although electron microscopy can harvest high-resolution images, its limited field of view restricts its analytical throughput. Increasing the magnification to improve confidence in identifying particle integrity further narrows the sampling field. Field selection in electron microscopy is highly operator-dependent. Due to the manual nature of the evaluation, a theoretically large number of fields of view would be required to accurately assess the average viral particle integrity of vaccine samples. However, the limited field of view imposes an unmanageable workload, introducing bias and reducing reproducibility.

Vaccine manufacturers have long been in search of a laboratory assay capable of providing a simple, real-time quantitative assessment of rabies particle integrity. Our team has been working hard to develop an immunological assay to meet the quality control of vaccine manufacturers, although this is a very challenging attempt. RV’s genome is a single-stranded negative-sense RNA encoding five major structural proteins[22], in which N binds tightly to the viral RNA to form a ribonucleoprotein complex, which is enclosed within a lipid envelope embedded with G spikes[23]. Therefore, the N protein inside the viral particle cannot be exposed in structurally intact rabies virus particle. Conversely, when rabies virus particles are damaged by external factors, and the disruption of the outer membrane leads to the exposure of the internal N protein. Based on the observations, we boldly hypothesized that the amount of exposed N inside rabies virus particles is inversely correlated with the structural integrity of rabies virus particles, and this integrity of rabies virus particles could be quantitatively assessed by measuring the level of exposed N protein in rabies virus particles.

To achieve the quantitation of virus particle integrity in quality control of rabies vaccine, we pioneered a novel detection mode targeting the exposed N protein within rabies virus particles. In this model, anti-G Mab was immobilized on the wells to capture rabies virus particles, while the labeled anti-N was used as a signal tracer for the quantitative analysis of exposed nucleoproteins in rabies virus particles. The results of antibody combination screening exceeded our expectations as they revealed notable differences in signals intensity across different combinations. Given that RN42 and RG56 each exhibited the highest binding fluorescence signal in monoclonal antibody screening, it was not surprising that their combination yielded the optimal pairing. RN44 also demonstrated a strong signal in monoclonal antibody screening, indicating its good affinity for the N protein. However, when RN44 was paired with other G Mabs, the combinations showed weak signal intensities. This makes it impossible to determine whether the observed signal strength is driven by antibody affinity or specific epitope recognition. Thus, RN42 and RG56 currently represent the most effective antibody pairing, and the potential for improved combinations in future studies cannot be excluded.

As a novel analytical approach for quality control of rabies vaccine, the initial challenge we encountered when developing the system was the configuration of calibration. In the absence of a national or international standard for exposed nucleoprotein within rabies virus particle, and considering that the exposed N content within rabies virus particle in the finished vaccine is theoretically higher than that in semi-finished vaccine products, we assigned a value of 1 EU/mL to the collaborative calibration of the 9th national standard for human rabies vaccine as a collaborative calibrator for this assay. Preliminary validation results suggest that the current calibrator is suitable for rabies vaccine samples at different stages of production within the same manufacturing process, but its applicability to rabies vaccine produced via alternative processes remains to be verified.

Due to the absence of comparable assay methodologies or reagents, the performance verification results obtained in this study cannot be directly benchmarked. However, the coefficients of variation in the precision analysis were all within 10%, demonstrating the high accuracy of the system. The accuracy analysis of freshly harvested, purified, and nearly expired purified virus particle samples yielded results consistent with expectations. Those rabies vaccine samples analyzed in the accuracy analysis test included almost all the sample types required for quality control, confirming that the performance of the current TRFIA fully meets the demands of vaccine quality control. Following the application of stress conditions such as ultrasonic crushing, repeated freeze-thawing, heat treatment and viral lysis to the finished vaccine, the current TRFIA effectively monitored changes in particle integrity in real time. With the exception of the repeated freeze-thaw experimental group, all treatment groups showed a consistent pattern: the detection signal first increased and then decreased, indicating a progressive loss of particle integrity until complete fragmentation led to a decline in signal. The observation that the detection signal of the repeated freeze-thaw experimental group increased without subsequent decline may be attributed to the fact that the HSA in the samples acted as an anti-freeze-thaw protection, preventing complete fragmentation of the viral particles. The current TRFIA results are consistent with the electron microscopy results, providing compelling evidence that N protein exposure within rabies virus particles serve as a reliable target for real-time quantitative assessment of virus particle integrity.

While the current TRFIA assay has proven to be a valuable tool in our study, certain limitations must be acknowledged. One notable limitation is the difficulty in differentiating between fully intact and highly ruptured rabies virus particles using the TRFIA assay alone. Although such occurrences are rare under the stringent quality standards of commercial vaccine production, recognizing this limitation remains essential. In addition, factors such as potential variations in antibody specificity and the subsequent need for re-evaluation in response to strain variation should also be considered. These limitations underscore the importance of using complementary techniques, such as electron microscopy, for confirmatory analysis when required.

To summarize, we successfully developed a TRFIA for real-time quantitative quality control analysis of rabies virus particle integrity in human rabies vaccine based on quantitative detection of exposed N within rabies virus particle. We look forward to the dissemination of this analytical approach will contribute to improvements in vaccine manufacturing processes, overall vaccine quality control and product characterization.

## Supporting information

S1 Table Results of serum potencies of mouse immunized with purified inactivated PM strain rabies virus. (DOCX)

S2 Table Result of antibody pairing screening. (DOCX)

S3 Table Selection of the optimal capture antibody concentration. (DOCX)

S4 Table Selection of the 0ptimal labeled antibody dilution. (DOCX)

S5 Table Selection of the optimal reaction time. (DOCX)

S6 Table Data of verification of design concept. (DOCX)

S7 Table Data of construction of standard curve. (DOCX)

S8 Table Sensitivity assay of the present TRFIA. (DOCX)

S9 Table Data of multiple vaccine samples detection. (DOCX)

S10 Table Data of finished vaccine sample (PM strain) treating with different artificially destroy. (DOCX)

S11 Table Data of Comparison with electron microscope observation. (DOCX)

S1 Fig Western-bolt results of RN42 and RG56. (TIF)
