# Supplementary material for: Exposed nucleoprotein inside rabies virus particle as an ideal target for real-time quantitative evaluation of rabies virus particle integrity in vaccine quality control
Source: PLoS Negl Trop Dis. 2025 May 30;19(5):e0013077. doi: 10.1371/journal.pntd.0013077 (PMC12124496; doi:10.1371/journal.pntd.0013077)
Supplement: S1 Table — (DOCX) [file pntd.0013077.s001.docx]

**S1 Table.** Results of serum potencies of mouse immunized with purified inactivated PM strain rabies virus.

| Dilution ratio | OD value (450nm) | | | | | | |
| --- | --- | --- | --- | --- | --- | --- | --- |
|  | ID of mouse | | | | | Positive control (Serum from mice immunized with rabies vaccine) | Negative control (PBS) |
|  | 1 | 2 | 3 | 4 | 5 |  |  |
| 5×10^-3^ | 2.94 | 3.06 | 2.83 | 2.76 | 3.01 | 2.603 | 0.05 |
| 1×10^-4^ | 2.652 | 2.43 | 2.387 | 2.361 | 2.827 | 1.25 | - |
| 2×10^-4^ | 2.266 | 1.51 | 1.52 | 1.2 | 2.493 | 0.51 | - |
| 4×10^-4^ | 1.35 | 0.99 | 0.71 | 0.62 | 2.174 | 0.21 | - |
| 12×10^-4^ | 0.38 | .039 | 0.269 | 0.35 | 0.79 | 0.12 | - |
