# Supplementary material for: Exposed nucleoprotein inside rabies virus particle as an ideal target for real-time quantitative evaluation of rabies virus particle integrity in vaccine quality control
Source: PLoS Negl Trop Dis. 2025 May 30;19(5):e0013077. doi: 10.1371/journal.pntd.0013077 (PMC12124496; doi:10.1371/journal.pntd.0013077)
Supplement: S2 Table — (DOCX) [file pntd.0013077.s002.docx]

**S2 Table.** Result of antibody pairing screening.

| Labeled antibody | Fluorescence intensity | | |
| --- | --- | --- | --- |
|  | Capture antibody | | |
|  | RG10 | RG18 | RG56 |
| RN03 | 22274 | 88234 | 69108 |
| RN19 | 54398 | 63544 | 49126 |
| RN42 | 111593 | 86397 | **186829** |
| RN44 | 12090 | 9837 | 18377 |
