# Supplementary material for: Exposed nucleoprotein inside rabies virus particle as an ideal target for real-time quantitative evaluation of rabies virus particle integrity in vaccine quality control
Source: PLoS Negl Trop Dis. 2025 May 30;19(5):e0013077. doi: 10.1371/journal.pntd.0013077 (PMC12124496; doi:10.1371/journal.pntd.0013077)
Supplement: S3 Table — (DOCX) [file pntd.0013077.s003.docx]

**S3 Table**. Selection of the optimal capture antibody concentration.

| Test batch | Fluorescence intensity | | | | |
| --- | --- | --- | --- | --- | --- |
|  | Concentration of capture antibody (μg/mL) | | | | |
|  | 0.5 | 1 | 3 | 5 | 8 |
| 1 | 20089 | 34511 | 94402 | 99584 | 101587 |
| 2 | 21454 | 38745 | 95221 | 99414 | 104541 |
| 3 | 23544 | 38784 | 96211 | 101010 | 100014 |
