# Supplementary material for: Exposed nucleoprotein inside rabies virus particle as an ideal target for real-time quantitative evaluation of rabies virus particle integrity in vaccine quality control
Source: PLoS Negl Trop Dis. 2025 May 30;19(5):e0013077. doi: 10.1371/journal.pntd.0013077 (PMC12124496; doi:10.1371/journal.pntd.0013077)
Supplement: S4 Table — (DOCX) [file pntd.0013077.s004.docx]

**S4 Table**. Selection of the optimal labeled antibody dilution.

| Test batch | Fluorescence intensity | | | | |
| --- | --- | --- | --- | --- | --- |
|  | Dilution of labeled antibody | | | | |
|  | 1:1600 | 1:800 | 1:400 | 1:200 | 1:100 |
| 1 | 18532 | 43543 | 101587 | 120014 | 130322 |
| 2 | 17645 | 42343 | 104541 | 116587 | 132221 |
| 3 | 16754 | 40321 | 100014 | 120541 | 129899 |
