# Supplementary material for: Exposed nucleoprotein inside rabies virus particle as an ideal target for real-time quantitative evaluation of rabies virus particle integrity in vaccine quality control
Source: PLoS Negl Trop Dis. 2025 May 30;19(5):e0013077. doi: 10.1371/journal.pntd.0013077 (PMC12124496; doi:10.1371/journal.pntd.0013077)
Supplement: S5 Table — (DOCX) [file pntd.0013077.s005.docx]

**S5 Table**. Selection of the optimal reaction time.

| Test batch | Fluorescence intensity | | | |
| --- | --- | --- | --- | --- |
|  | Reaction time (min) | | | |
|  | 20 | 40 | 60 | 80 |
| 1 | 18532 | 77543 | 101587 | 99014 |
| 2 | 17645 | 76343 | 104541 | 106587 |
| 3 | 16754 | 80321 | 100014 | 105541 |
