# Supplementary material for: Exposed nucleoprotein inside rabies virus particle as an ideal target for real-time quantitative evaluation of rabies virus particle integrity in vaccine quality control
Source: PLoS Negl Trop Dis. 2025 May 30;19(5):e0013077. doi: 10.1371/journal.pntd.0013077 (PMC12124496; doi:10.1371/journal.pntd.0013077)
Supplement: S6 Table — (DOCX) [file pntd.0013077.s006.docx]

**S6 Table**. Data of verification of design concept.

| Combination | Concentration of virus lysis buffer (100%) | Fluorescence intensity | | |
| --- | --- | --- | --- | --- |
|  |  | 1 | 2 | 3 |
| Coated G-MAb&Labeled G-MAb | 0 | 2497704 | 2420373 | 2445415 |
|  | 5 | 1956437 | 1902848 | 1851168 |
|  | 10 | 1506654 | 1516254 | 1609673 |
|  | 25 | 730488 | 727939 | 771906 |
|  | 50 | 686939 | 598035 | 631209 |
| Coated G-MAb&Labeled N-MAb | 0 | 34935 | 32547 | 31480 |
|  | 5 | 267891 | 295885 | 275269 |
|  | 10 | 323488 | 312091 | 295806 |
|  | 25 | 189943 | 174325 | 176065 |
|  | 50 | 133268 | 120822 | 118224 |
| Coated N-MAb&Labeled N-MAb | 0 | 64463 | 67380 | 63947 |
|  | 5 | 64798 | 72529 | 67114 |
|  | 10 | 124901 | 136234 | 136898 |
|  | 25 | 151615 | 140056 | 154009 |
|  | 50 | 164380 | 154044 | 149835 |
