# Supplementary material for: Exposed nucleoprotein inside rabies virus particle as an ideal target for real-time quantitative evaluation of rabies virus particle integrity in vaccine quality control
Source: PLoS Negl Trop Dis. 2025 May 30;19(5):e0013077. doi: 10.1371/journal.pntd.0013077 (PMC12124496; doi:10.1371/journal.pntd.0013077)
Supplement: S7 Table — (DOCX) [file pntd.0013077.s007.docx]

**S7 Table**. Data of construction of standard curve.

| Standard (mEU/ml) | Fluorescence intensity | | | | | | | | | |
| --- | --- | --- | --- | --- | --- | --- | --- | --- | --- | --- |
|  | 1 | 2 | 3 | 4 | 5 | 6 | 7 | 8 | 9 | 10 |
| 0 | 1860 | 2320 | 2261 | 2235 | 2077 | 2149 | 1924 | 2092 | 2066 | 2263 |
| 2.5 | 9819 | 9345 | 9788 | 9218 | 8518 | 9514 | 9973 | 8317 | 8193 | 9626 |
| 12.5 | 42329 | 36396 | 40611 | 36704 | 34855 | 39129 | 36912 | 35030 | 37906 | 37028 |
| 25 | 69078 | 63784 | 70687 | 63502 | 66703 | 66264 | 70933 | 68996 | 61192 | 77896 |
| 50 | 134574 | 127941 | 119621 | 132983 | 123431 | 122793 | 134234 | 134737 | 123487 | 120351 |
| 125 | 249556 | 261676 | 254960 | 254024 | 290600 | 249396 | 245557 | 250692 | 248388 | 254517 |
