# Supplementary material for: Exposed nucleoprotein inside rabies virus particle as an ideal target for real-time quantitative evaluation of rabies virus particle integrity in vaccine quality control
Source: PLoS Negl Trop Dis. 2025 May 30;19(5):e0013077. doi: 10.1371/journal.pntd.0013077 (PMC12124496; doi:10.1371/journal.pntd.0013077)
Supplement: S8 Table — (DOCX) [file pntd.0013077.s008.docx]

**S8 Table**. Sensitivity assay of the present TRFIA.

| ID | Fluorescence intensity |
| --- | --- |
| 1 | 2933 |
| 2 | 2206 |
| 3 | 2337 |
| 4 | 2640 |
| 5 | 2320 |
| 6 | 2528 |
| 7 | 2492 |
| 8 | 2252 |
| 9 | 2448 |
| 10 | 2831 |
| 11 | 2235 |
| 12 | 2670 |
| 13 | 2241 |
| 14 | 2221 |
| 15 | 2244 |
| 16 | 2229 |
| 17 | 2488 |
| 18 | 2460 |
| 19 | 2130 |
| 20 | 2200 |
| Mean+2×SD | 2853 |
