# Supplementary material for: Exposed nucleoprotein inside rabies virus particle as an ideal target for real-time quantitative evaluation of rabies virus particle integrity in vaccine quality control
Source: PLoS Negl Trop Dis. 2025 May 30;19(5):e0013077. doi: 10.1371/journal.pntd.0013077 (PMC12124496; doi:10.1371/journal.pntd.0013077)
Supplement: S9 Table — (DOCX) [file pntd.0013077.s009.docx]

| Sample | Value of glycoprotein (IU/mL) | Value of exposed N (EU/mL) |
| --- | --- | --- |
| Virus culture sample | 9.3 | 0.1 |
|  | 9.5 | 0.15 |
|  | 9.45 | 0.1 |
| Virus culture sample | 9.6 | 0.15 |
|  | 9.5 | 0.2 |
|  | 9.4 | 0.17 |
| Virus purification sample | 9.2 | 0.49 |
|  |  | 0.48 |
|  | 9.1 | 0.48 |
|  | 8.9 | 0.49 |
|  |  | 0.55 |
| Virus purification sample | 9.2 | 0.44 |
|  |  | 0.44 |
|  | 9 | 0.405 |
|  | 9.1 | 0.46 |
|  |  | 0.475 |
| Nearly expired virus purification sample | 9.7 | 1.44 |
|  |  | 1.305 |
|  | 9.5 | 1.32 |
|  | 9.6 | 1.36 |
|  |  | 1.47 |

**S9 Table**. Data of multiple vaccine samples detection.
