# Supplementary material for: Exposed nucleoprotein inside rabies virus particle as an ideal target for real-time quantitative evaluation of rabies virus particle integrity in vaccine quality control
Source: PLoS Negl Trop Dis. 2025 May 30;19(5):e0013077. doi: 10.1371/journal.pntd.0013077 (PMC12124496; doi:10.1371/journal.pntd.0013077)
Supplement: S10 Table — (DOCX) [file pntd.0013077.s010.docx]

**S10 Table**. Data of finished vaccine sample (PM strain) treating with different artificially destroy.

| Test batch | Value of exposed N (EU/mL) | | | | | | | | | | | | | | | | | | | | | | | | | |
| --- | --- | --- | --- | --- | --- | --- | --- | --- | --- | --- | --- | --- | --- | --- | --- | --- | --- | --- | --- | --- | --- | --- | --- | --- | --- | --- |
|  | Time of ultrasound treatment (min) | | | | | Times of repeated freezing and thawing | | | | | | | | Time of heat treatment (h) | | | | | | | | | Treat time of 0.5% Triton X-100 (h) | | | |
|  | 0 | 1 | 10 | 30 | 120 | 0 | 1 | 3 | 5 | 7 | 10 | 15 | 20 | 0 | 0.16 | 0.33 | 0.5 | 0.66 | 0.83 | 1 | 8 | 24 | 0 | 4 | 8 | 24 |
| 1 | 0.49 | 0.6 | 2.15 | 2.51 | 1.55 | 0.5 | 0.53 | 0.54 | 0.55 | 0.59 | 0.64 | 0.95 | 1.48 | 0.51 | 0.68 | 0.56 | 0.46 | 0.4 | 0.39 | 0.32 | 0.08 | 0.06 | 0.51 | 3.51 | 5.98 | 1.56 |
| 2 | 0.53 | 0.55 | 2.25 | 2.65 | 1.45 | 0.51 | 0.52 | 0.53 | 0.57 | 0.56 | 0.7 | 0.92 | 1.44 | 0.5 | 0.65 | 0.55 | 0.47 | 0.39 | 0.38 | 0.3 | 0.09 | 0.05 | 0.52 | 3.98 | 5.56 | 1.98 |
