# Supplementary material for: Exposed nucleoprotein inside rabies virus particle as an ideal target for real-time quantitative evaluation of rabies virus particle integrity in vaccine quality control
Source: PLoS Negl Trop Dis. 2025 May 30;19(5):e0013077. doi: 10.1371/journal.pntd.0013077 (PMC12124496; doi:10.1371/journal.pntd.0013077)
Supplement: S11 Table — (DOCX) [file pntd.0013077.s011.docx]

**S11 Table**. Data of Comparison with electron microscope observation.

| Sample | Value of exposed N (EU/mL) |
| --- | --- |
| Untreated sample | 0.24 |
|  | 0.22 |
|  | 0.23 |
| Treated sample | 0.77 |
|  | 0.76 |
|  | 0.72 |
